# Supplementary figures and images for: Phytoplasma-induced alterations in endophytic bacterial communities in Paulownia: implications for witches’ broom
Source: Microbiol Spectr. 2025 Sep 11;13(10):e01489-25. doi: 10.1128/spectrum.01489-25 (PMC12502696; doi:10.1128/spectrum.01489-25)

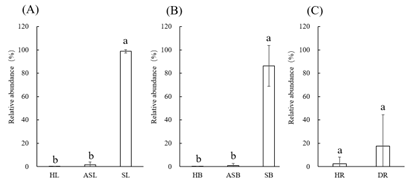

Supplement: Fig. S1 — Abundances of Phytoplasma in the different compartments of Paulownia. [file spectrum.01489-25-s0001.tif]

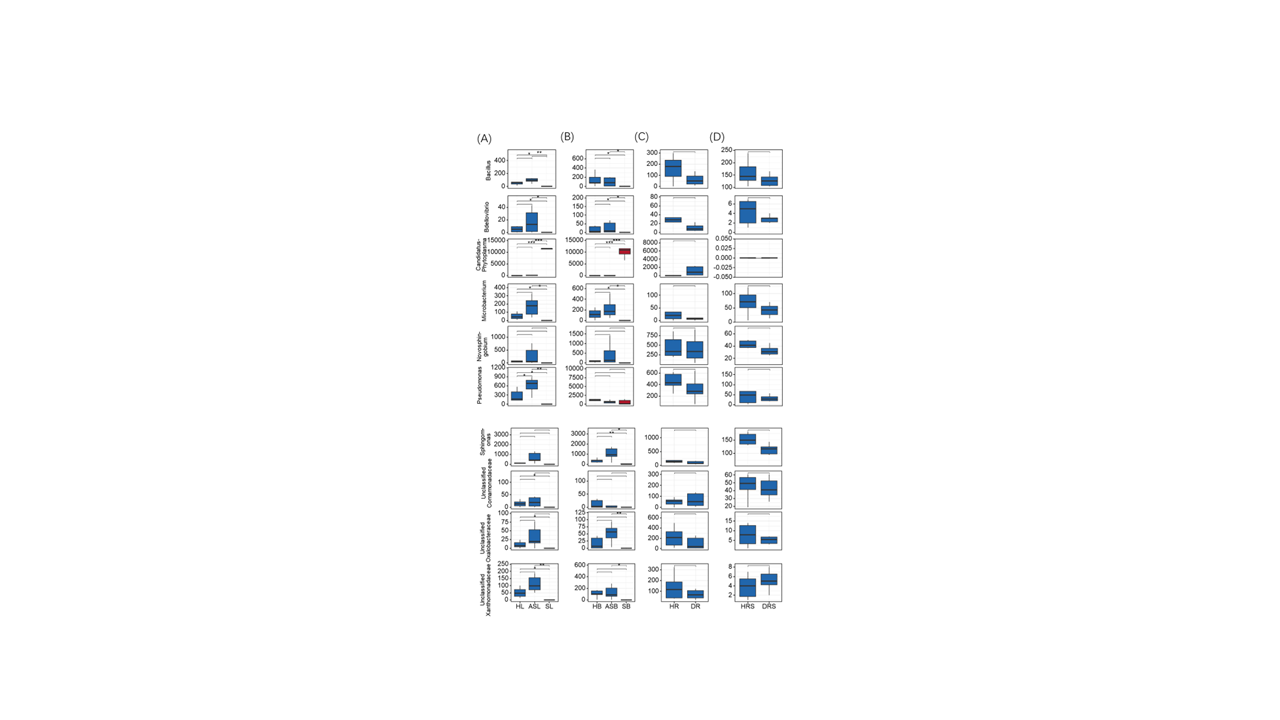

Supplement: Fig. S2 — Abundance comparison of biomarkers in congeneric samples (reads). [file spectrum.01489-25-s0002.tif]

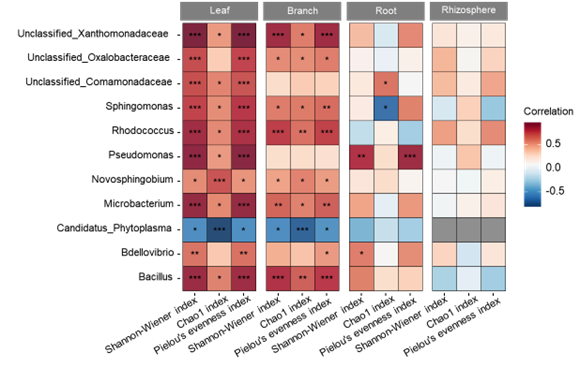

Supplement: Fig. S3 — Spearman correlation analysis between biomarkers and bacteria α diversity. [file spectrum.01489-25-s0003.tif]

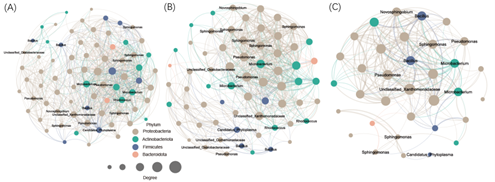

Supplement: Fig. S4 — Bacterial network interactions in samples with different symptoms. [file spectrum.01489-25-s0004.tif]

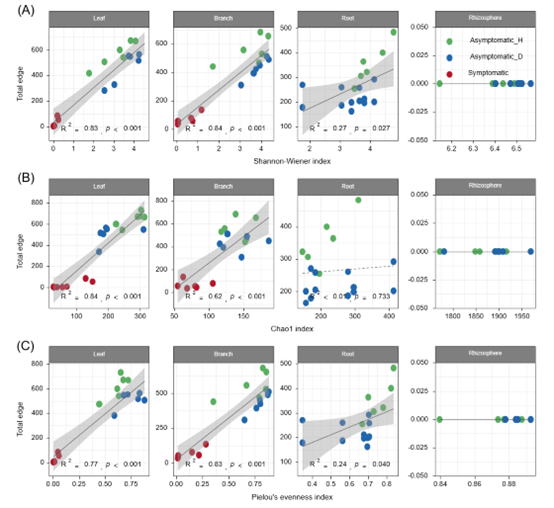

Supplement: Fig. S5 — Linear fitting analysis of bacterial community α diversity and network complexity. [file spectrum.01489-25-s0005.tif]

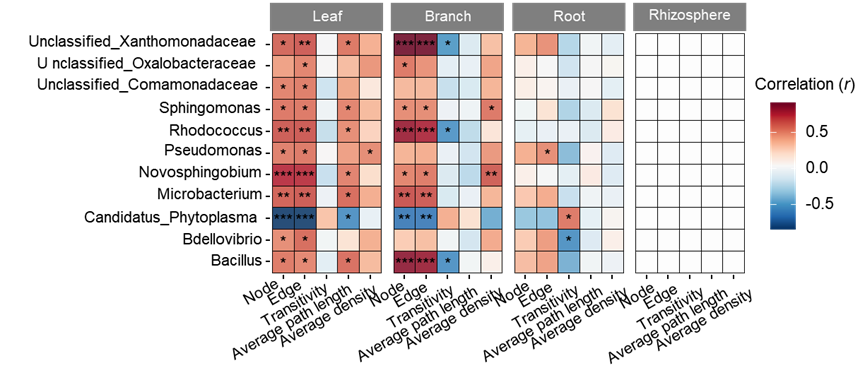

Supplement: Fig. S6 — Spearman correlation analysis between biomarkers and network topological parameters in different samples. [file spectrum.01489-25-s0006.tif]

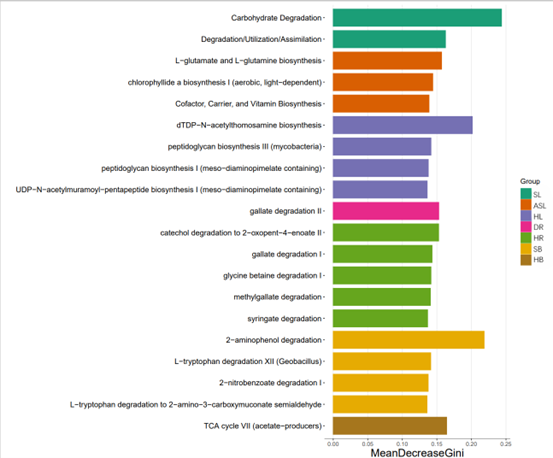

Supplement: Fig. S7 — PICRUSt2 analysis of metabolic pathways of key metabolic pathway gene abundances in diseased vs. healthy tissues in Paulownia. [file spectrum.01489-25-s0007.tif]

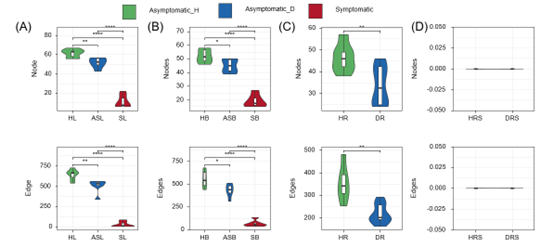

Supplement: Fig. S8 — Comparison of the number of edges and nodes in bacterial community networks among different samples. [file spectrum.01489-25-s0008.tif]
