# Supplementary material for: Phytoplasma-induced alterations in endophytic bacterial communities in Paulownia: implications for witches’ broom
Source: Microbiol Spectr. 2025 Sep 11;13(10):e01489-25. doi: 10.1128/spectrum.01489-25 (PMC12502696; doi:10.1128/spectrum.01489-25)
Supplement: Supplemental material — Supplemental figure captions. [file spectrum.01489-25-s0009.docx]

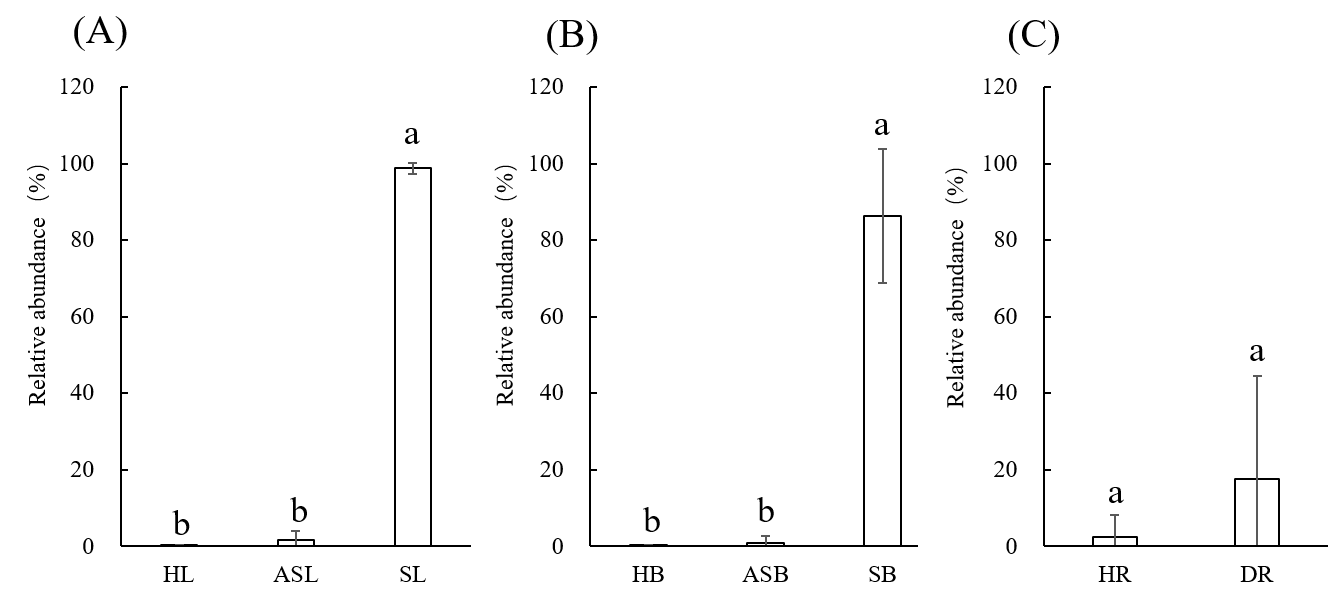


**Fig. S1** Abundances of Phytoplasma in the different compartments of Paulownia. Different lowercase letters in the same part of figure indicate statistically significant differences between treatments as determined by one-way ANOVA with post hoc Tukey HSD test (*P*<0.05). (A: Leaves; B: Branches; C: Roots; HB: Healthy branch; HL: Healthy leaf; HR: Healthy root; HRS: Healthy rhizosphere soil; SB: Symptomatic branch; SL: Symptomatic leaf; ASB: Asymptomatic branch; ASL: Asymptomatic leaf; DR: Diseased root; DRS: Diseased rhizosphere soil)


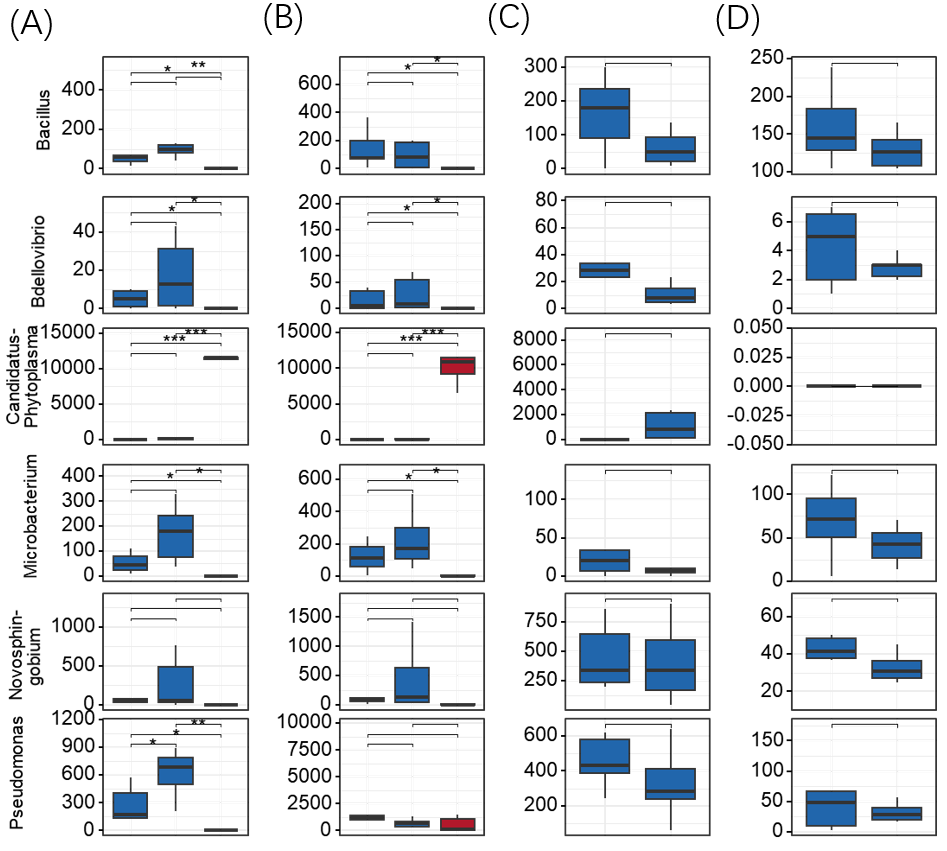


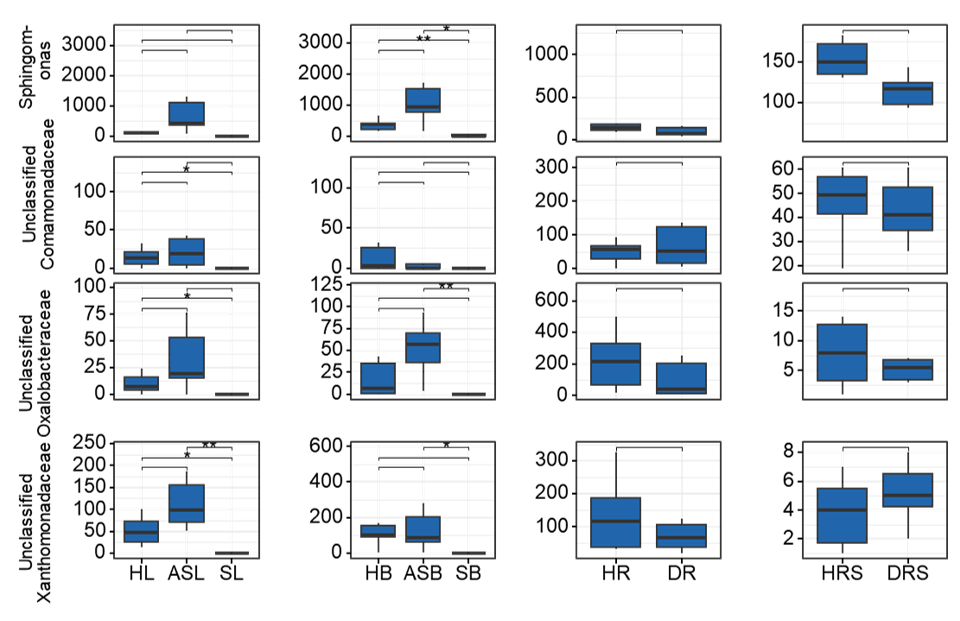


**Fig. S2** Abundance comparison of biomarkers in congeneric samples (reads)。* represented *P*<0.05; ** represented *P*<0.01; *** represented *P*<0.001 (HB: Healthy branch; HL: Healthy leaf; HR: Healthy root; HRS: Healthy rhizosphere soil; SB: Symptomatic branch; SL: Symptomatic leaf; ASB: Asymptomatic branch; ASL: Asymptomatic leaf; DR: Diseased root; DRS: Diseased rhizosphere soil)


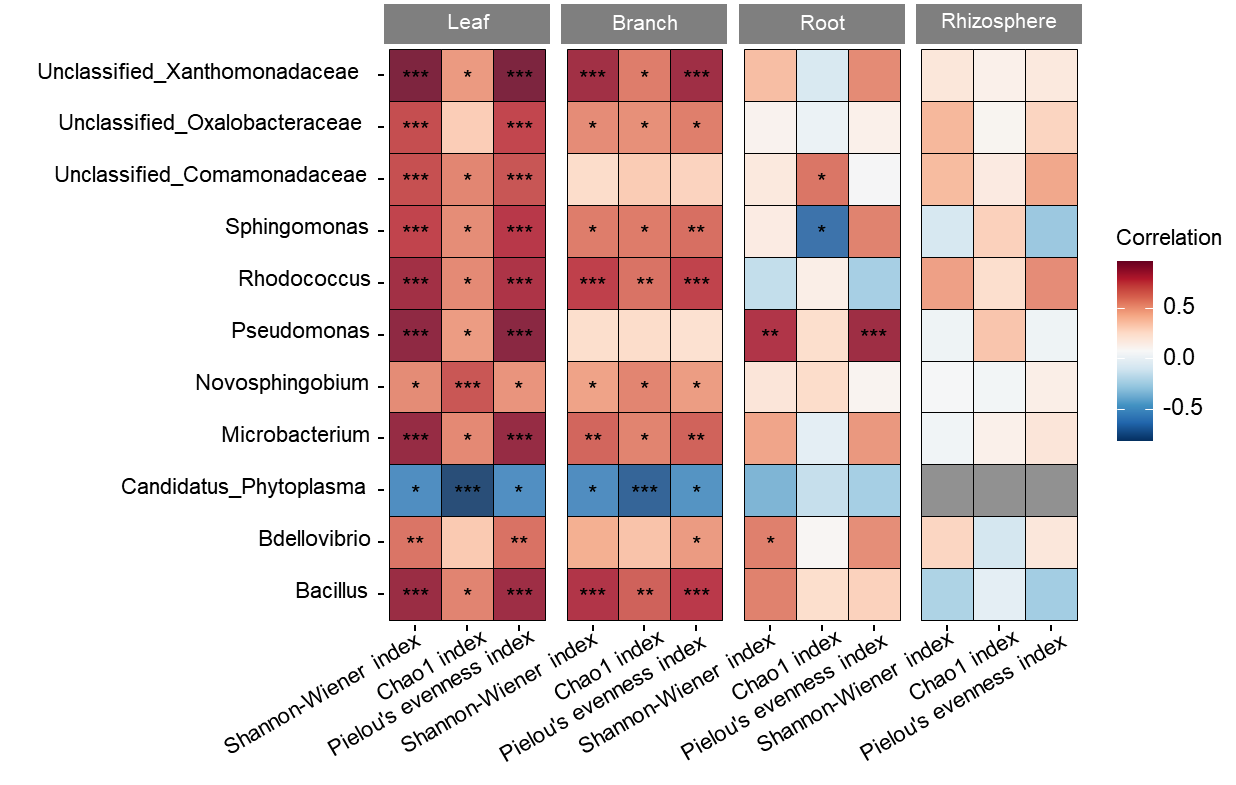


**Fig. S3** Spearman correlation analysis between biomarkers and bacteria α diversity. Red represents a positive correlation; Blue represents the negative correlation. The color intensity reflects the strength and direction of these correlation. *represent *P*<0.05; ** represent *P*<0.01; *** represent *P*<0.001


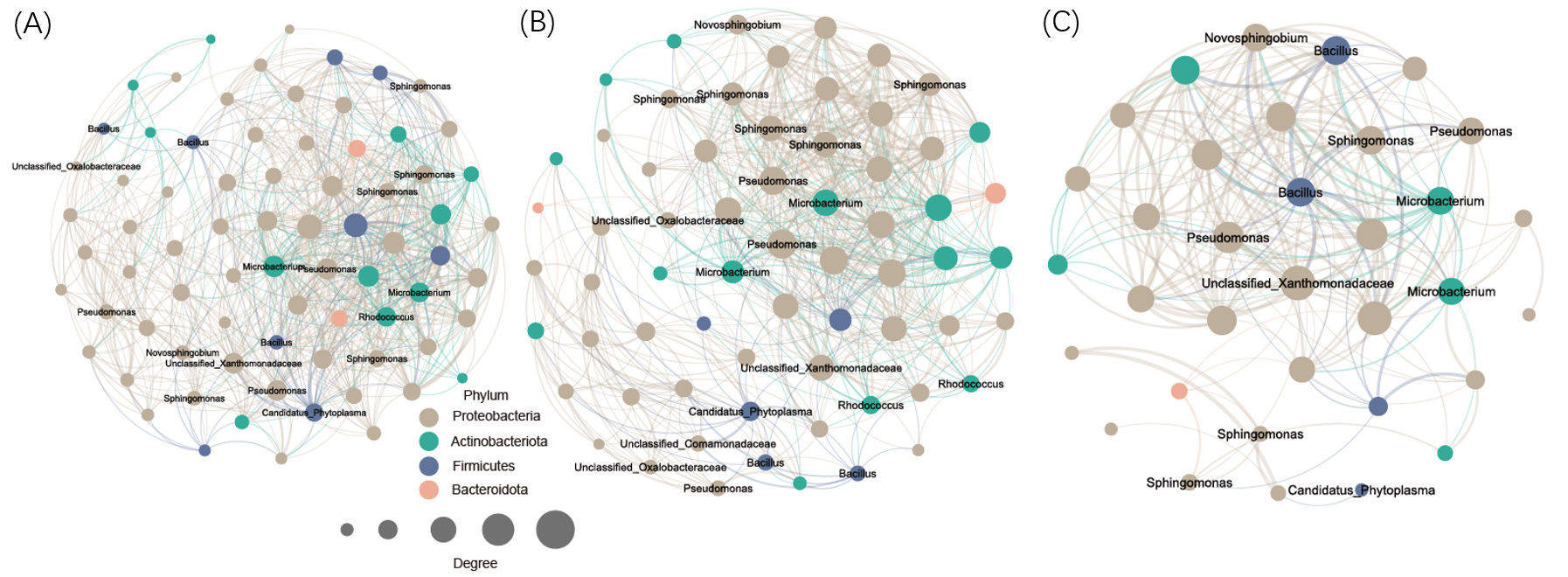


**Fig. S4** Bacterial network interactions in samples with different symptoms (A contained healthy branches, leaves and roots; B contained asymptomatic branches, leaves and diseased roots; C contained symptomatic branches, leaves and diseased roots)


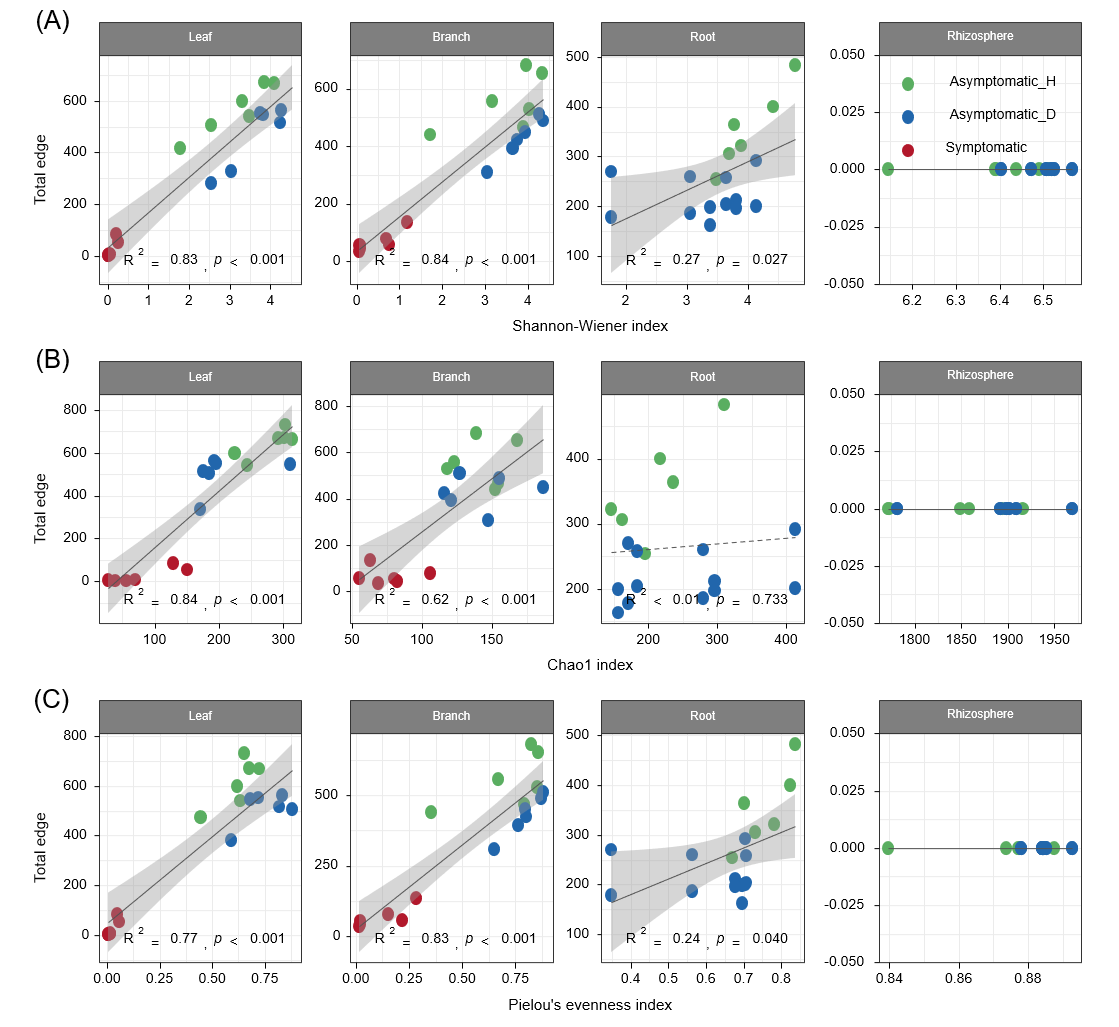


**Fig. S5** Linear fitting analysis of bacterial community α diversity and network complexity (**number of edges**) (A: Shannon-Wiener indices; B: Chao1 indices; C: Pielou’s evenness indices)


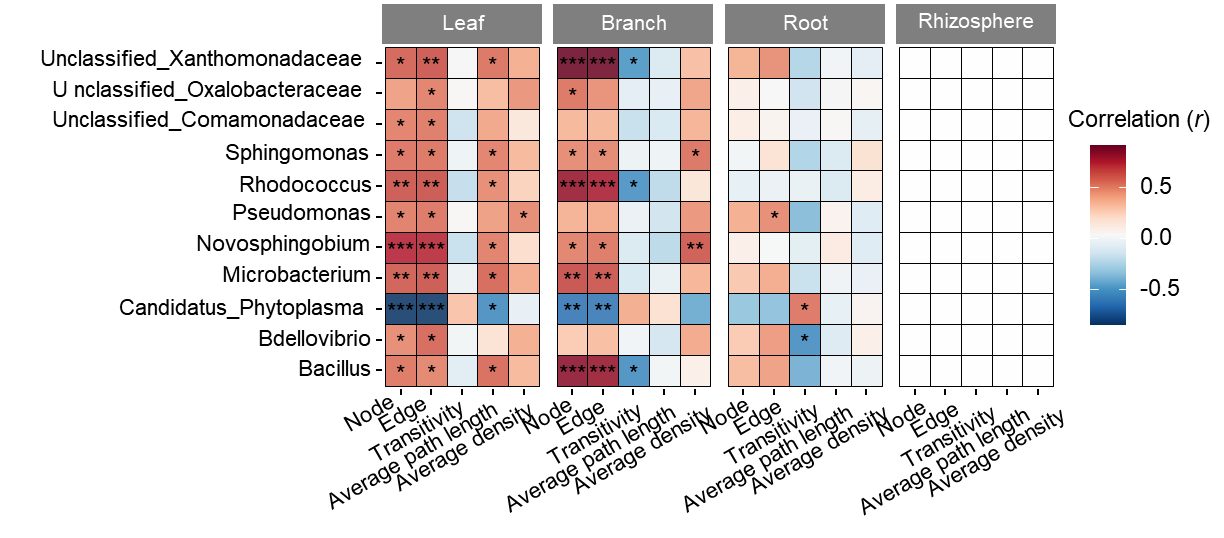


**Fig. S**6 Spearman correlation analysis between biomarkers and network topological parameters in different samples. Blue represented negative correlation, red represented positive correlation. * represented *P*<0.05; ** represented *P*<0.01; *** represented *P*<0.001


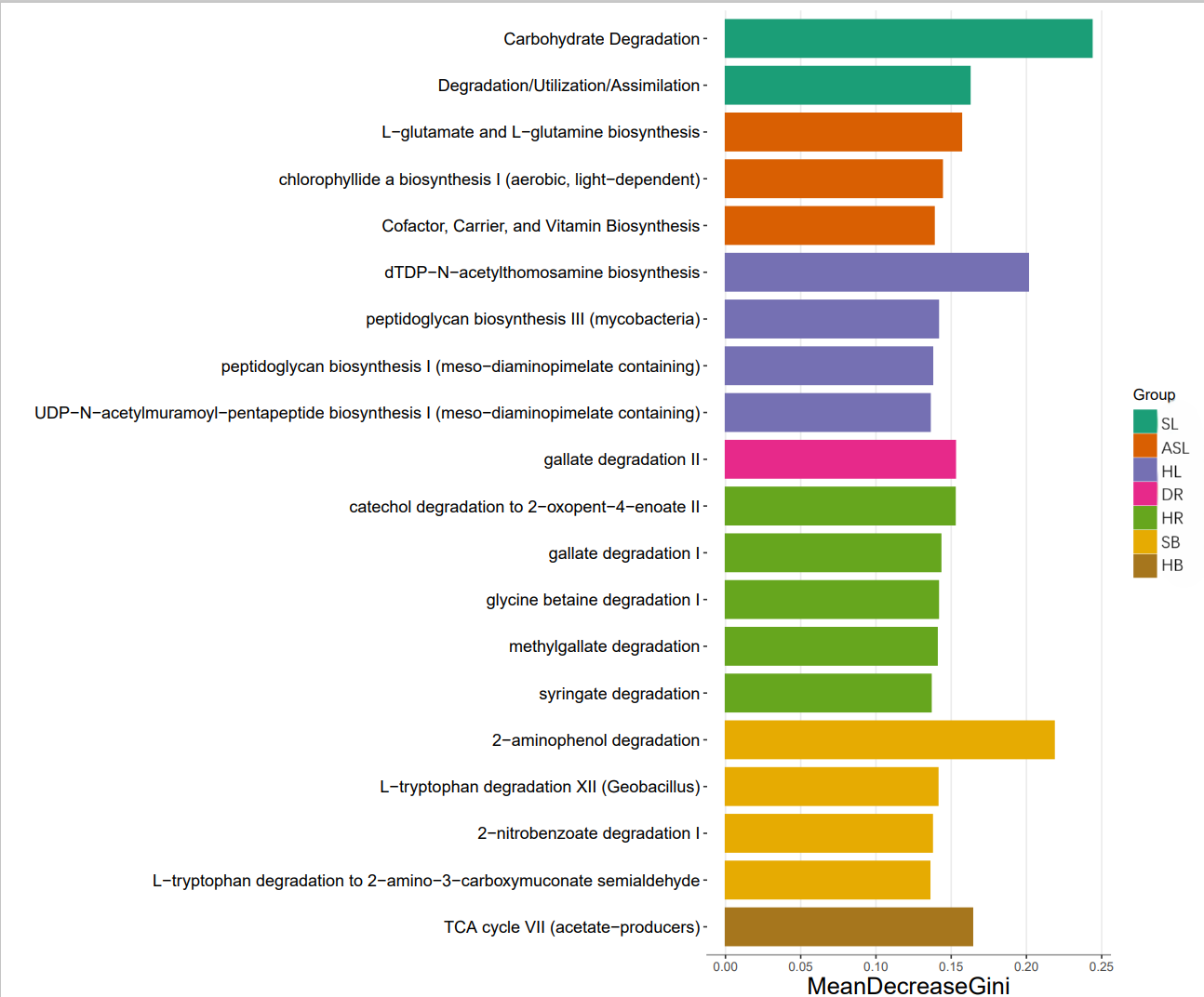


**Fig. S7** PICRUSt2 analysis of metabolic pathways of key metabolic pathway gene abundances in diseased vs. healthy tissues in Paulownia. SL: Symptomatic leaf; ASL: Asymptomatic leaf; HL: Healthy leaf; DR: Diseased root; HR: Healthy root; SB: Symptomatic branch; HB: Healthy branch;


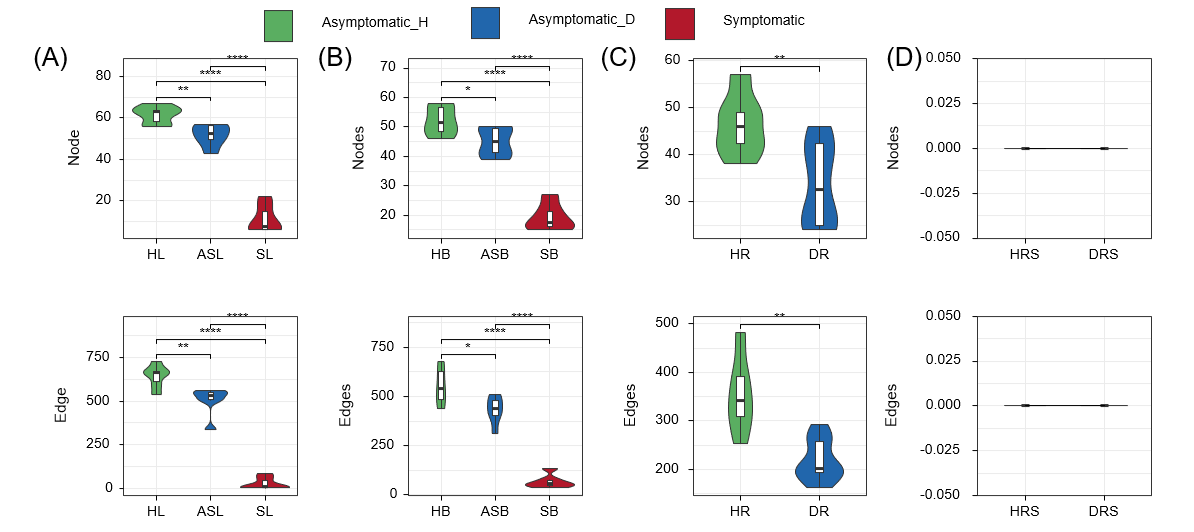


**Fig. S8** Comparison of the number of edges and nodes in bacterial community networks among different samples (A: Leaves; B: Branches; C: roots; D: Rhizosphere soil; HB: Healthy branch; HL: Healthy leaf; HR: Healthy root; HRS: Healthy rhizosphere soil; SB: Symptomatic branch; SL: Symptomatic leaf; ASB: Asymptomatic branch; ASL: Asymptomatic leaf; DR: Diseased root; DRS: Diseased rhizosphere soil)
