# Supplementary material for: Phytoplasma-induced alterations in endophytic bacterial communities in Paulownia: implications for witches’ broom
Source: Microbiol Spectr. 2025 Sep 11;13(10):e01489-25. doi: 10.1128/spectrum.01489-25 (PMC12502696; doi:10.1128/spectrum.01489-25)
Supplement: Supplemental tables — Tables S1 to S5. [file spectrum.01489-25-s0010.docx]

**Table S1**. Primer Information for Bacterial Community Analysis

| **Target Region** | **Forward Primer (5′–3′)** | **Reverse Primer (5′–3′)** |
| --- | --- | --- |
| V5–V7 | 799F: AACMGGATTAGATACCCKG | 1193R: ACGTCATCCCCACCTTCC |

**Table S2**

(A) PCR reaction system

| Component | Primary PCR (R16mF1/R16mR1) | Nested PCR (R16F2/R16R2) | Final Concentration |
| --- | --- | --- | --- |
| 5× PCR Buffer (with MgCl₂) | 5 µL | 5 µL | 1× |
| dNTPs (10 mM) | 0.5 µL | 0.5 µL | 0.2 mM |
| Forward Primer (10 µM) | 1 µL | 1 µL | 0.4 µM |
| Reverse Primer (10 µM) | 1 µL | 1 µL | 0.4 µM |
| Taq DNA Polymerase (5 U/µL) | 0.2 µL | 0.2 µL | 1 U |
| Template DNA | 2 µL (genomic DNA) | 1 µL (1:20 dilution) |  |
| Nuclease-free H₂O | 15.3 µL | 16.3 µL |  |
| Total Volume | 25 µL | 25 µL |  |

(B) PCR Thermal Cycling Profile

| Step | Temperature | Duration | Cycles |
| --- | --- | --- | --- |
| Initial Denaturation | 94 °C | 3 min | 1 |
| Denaturation | 94 °C | 1 min | 5 (for the first block) |
| Annealing | 52 °C | 1 min |  |
| Extension | 72 °C | 2 min |  |
| Denaturation | 94 °C | 30 s | 25 (for the second block) |
| Annealing | 52 °C | 1 min |  |
| Extension | 72 °C | 2 min |  |
| Final Extension | 72 °C | 10 min | 1 |

**Table S3** primer pairs of phytoplasma gene

| Primer name | Primer sequence |
| --- | --- |
| R16mF1 | 5’- CAT GCA AGT CGA ACG GA -3’ |
| R16mR1 | 5’- CTT AAC CCC AAT CAT CGA -3’ |
| R16F2 | 5’- ACG ACT GCT AAG ACT GG -3’ |
| R16R2 | 5’- GCG GTG TGT ACA AAC CCC G -3’ |

**Table S4 PCR reaction system**

| Reagent | Volume |
| --- | --- |
| 10× Buffer | 2.0 μL |
| 2.5 mM dNTPs | 2.0 μL |
| Forward Primer（5 μM） | 0.8 μL |
| Reverse Primer（5 μM） | 0.8 μL |
| rTaq Polymerase | 0.2 μL |
| BSA（20 mg/ml） | 0.2 μL |
| Template DNA | 10.0 ng |
| ddH_2_O | Up to 20.0 μL |

**Table S5** Parameters of bacterial co-occurrence networks along the different of compartments of *Paulownia* plant

| **Samples^*^** | **Node** | **Total edge** | **Positive edge / %** | **Negative edge / %** | **Average degree** | **Average clustering**  **coefficient** | **Average path**  **distance** |
| --- | --- | --- | --- | --- | --- | --- | --- |
| HL | 158 | 252 | 62.7 | 37.3 | 3.190 | 0.518 | 9.016 |
| ASL | 94 | 138 | 53.6 | 46.4 | 2.936 | 0.434 | 3.848 |
| SL | 14 | 16 | 75.0 | 25.0 | 2.286 | 1.000 | 1.000 |
| HB | 66 | 90 | 75.6 | 24.4 | 2.727 | 0.483 | 3.676 |
| ASB | 68 | 97 | 62.9 | 37.1 | 2.853 | 0.504 | 4.728 |
| SB | 19 | 21 | 90.5 | 9.5 | 2.211 | 0.583 | 1.471 |
| HR | 126 | 282 | 77.7 | 22.3 | 4.476 | 0.607 | 7.980 |
| DR | 119 | 342 | 78.4 | 21.6 | 5.748 | 0.626 | 5.936 |
| HRS | 421 | 2118 | 59.4 | 40.6 | 10.062 | 0.509 | 5.211 |
| DRS | 411 | 1780 | 56.4 | 43.6 | 8.662 | 0.512 | 5.603 |

*HB: Healthy branches; HL: Health leaves; HR: Health roots; HRS: Rhizosphere soil of health plant; SB: Symptomatic branches; SL: Symptomatic leaves; ASB: Asymptomatic branches; ASL: Asymptomatic leaves; DR: Diseased roots; DRS: Rhizosphere soil of disease plant
